# SIRT2 enhances 1-methyl-4-phenyl-1,2,3,6-tetrahydropyridine (MPTP)-induced nigrostriatal damage via apoptotic pathway

**DOI:** 10.3389/fnagi.2014.00184

**Published:** 2014-08-11

**Authors:** Lei Liu, Anirudh Arun, Lakia Ellis, Carina Peritore, Gizem Donmez

**Affiliations:** Department of Neuroscience, Tufts University School of MedicineBoston, MA, USA

**Keywords:** Parkinson's Disease, SIRT2, sirtuin, MPTP, apoptosis

## Abstract

Sirtuins are NAD-dependent protein deacetylases that were shown to have protective effects against different age-related diseases. SIRT2 is a strong deacetylase that is highly expressed in brain. It has been associated with neurodegenerative diseases. MPTP (1-methyl-4-phenyl-1,2,3,6-tetrahydropyridine) is a dopaminergic neurotoxin that displays clinical features of Parkinson's Disease (PD). MPTP leads to the degeneration of nigrostriatal dopaminergic pathway after its systemic administration. Chronic administration of MPTP induces lesion via apoptosis. We show here that SIRT2 deacetylates Foxo3a, increases RNA and protein levels of Bim, and as a result enhances apoptosis in the MPTP model of PD. We also show that neurodegeneration induced by chronic MPTP regimen is prevented by genetic deletion of SIRT2 in mouse. Deletion of SIRT2 leads to the reduction of apoptosis due to an increase in acetylation of Foxo3a and a decrease in Bim levels. We demonstrate that SIRT2 deacetylates Foxo3a, activates Bim, and induces apoptosis only in MPP^+^-treated cells. Therefore, designing SIRT2 inhibitors might be helpful in developing effective treatments for PD.

## Introduction

Parkinson's Disease (PD), the most common movement disorder and the second most common neurodegenerative disease after Alzheimer's disease, is characterized primarily by the loss of dopaminergic neurons. MPTP is the most commonly used dopaminergic neurotoxin that leads to the degeneration of nigrostriatal dopaminergic pathway after its systemic administration. It is still the only PD model that displays reproducible neurodegeneration. The chronic MPTP regimen leads to neurodegeneration via apoptosis (Dauer and Przedborski, 2003).

Sirtuins are NAD-dependent protein deacetylases that were shown to have protective effects against different age-related diseases (Donmez and Guarente, 2010; Donmez, 2012). SIRT1 was shown to reduce Abeta peptide formation in an Alzheimer's disease mouse model and suppress alpha-synuclein aggregation in A53T alpha-synuclein mouse model (Donmez et al., 2010, 2012). SIRT2 is a strong protein deacetylase, and is highly expressed in brain. It was shown to be an abundant neuronal protein that accumulates in the central nervous system of aging mice (Maxwell et al., 2011). SIRT2 was shown to co-localize with microtubules and functions as alpha-tubulin deacetylase. During G2/M phase, SIRT2 proteins enter nuclei and deacetylate histones (Donmez, 2012). A recent study showed that SIRT2 inhibitors protected against dopaminergic cell death both *in vitro* and in a *Drosophila* model of PD (Outerio et al., 2007). In addition, SIRT2 inhibition was shown to protect against Huntington's disease by reducing sterol biosynthesis (Luthi-Carter et al., 2010). SIRT2 expression was also shown to increase in cells with oxidative stress, such as hydrogen peroxide treatment and SIRT2 was shown to promote cell death when cells are under severe stress by activating Bim, a proapoptotic factor (Wang et al., 2007). However, the functional role and the effect of SIRT2 in brain and in a mouse model of a neurodegenerative disease have not been shown.

Here, we report that SIRT2 deacetylates Foxo3a and activates Bim in MPTP-treated (chronic regimen) mouse brains, inducing apoptotic neuronal death. We also show that in MPP^+^-treated SH-SY5Y cells, SIRT2 induces caspase-3 activated apoptotic cell death. MPTP-induced nigrostriatal damage is reduced in SIRT2 knockout (KO) mice, indicating that SIRT2 deletion is protective against this treatment by preventing apoptosis. In addition, silencing SIRT2 reduces and overexpressing SIRT2 increases caspase-3 activity in MPP^+^-treated SH-SY5Y cells. We show that SIRT2 deacetylates Foxo3a, activates Bim, and induces apoptosis only in MPP^+^-treated cells and only in MPTP-injected mouse brains. Therefore, we show here for the first time that inhibiting SIRT2 in a mouse model of PD might be protective against this disease and helpful in designing effective treatments in the future.

## Experimental procedures

### Mouse strain

All mice used were in congenic C57Bl/6. SIRT2 KO mice were generated by the targeted insertion of a puromycin resistance gene into exon 11 of the SIRT2 locus. The insertion introduces a stop codon that should result in nonsense-mediated decay of the SIRT2 mRNA (Bobrowska et al., 2012). All mice were housed at controlled temperature (25°C) and 12:12 h light-dark cycle. Mice used were littermates or their parents were littermates.

### Plasmids

The plasmids expressing mSIRT2 (13813) and Bim (8804) were purchased from Addgene. The SIRT2-shRNA (RMM3981-9579810) and Bim-shRNA (RHS4533-NM006538) plasmids were purchased from Open Biosystems.

### Immunohistochemistry

Mice were perfused with 4% paraformaldehyde, cryoprotected, and sectioned 30 μm-thick. Eight to twelve sections per brain were analyzed. Vectastain kit (Vector laboratories) was used to perform TH-staining according to manufacturer's directions using TH antibody (EMD-Millipore). Nissl staining was performed according to the manufacturer's protocol (IHCWORLD). TH-positive and Nissl-stained neurons in SNpc were counted by the image analysis tool of NIS-Elements AR microscope software. TH-positive striatal fibers were assessed by optical density. MPTP i.p. (intraperitoneal injection)-administered mice that were used for immunohistochemistry were 2 months old and received MPTP (30 mg kg^−1^ free base MPTP) daily for six consecutive days (Tatton and Kish, 1997; Jackson-Lewis and Przedborski, 2007).

### Western blotting and immunoprecipitation

Mouse brains were homogenized in RIPA buffer (50 mM Tris-HCl pH: 8.0, 1mM EDTA, 0.1% SDS, 150 mM NaCl, 1% NP40, 0.1% Sodium-deoxycholate) including Complete Protease Inhibitor mixture (Roche), centrifuged, 100 μg of the supernatant was loaded onto 4–15% gradient SDS-PAGE gels and immunoblotted with anti-SIRT2 (Cell signaling-12672, conc. 1:1000), Foxo3a (Abcam-ab47409, conc. 1:1000), Bim (Abcam-ab7888, conc. 1:1000), actin (Millipore-MAB1501, conc. 1:5000), Ac-K (Immunechem-ICP0380, conc.1:500) antibodies. Different SIRT2 antibodies were tested for specificity and Cell Signaling-12672 was used in the study (Supplementary Figure 1). Western blotting experiments were performed with at least six mice from each genotype and age and the representative blots are shown. For western blotting using cell extracts, cells were harvested and extracted in RIPA buffer as explained above. The immunoprecipitations were carried out by using Pierce Direct IP Kit (Thermo Scientific) according to manufacturer's directions. MPTP-administered mice that were used for western blotting and qPCR analysis were 3 months old and received 5 days of MPTP treatment.

### RNA isolation and analysis

Total RNA from mice brains or cells was isolated by using Trizol (Invitrogen). For real time q-PCR analysis, cDNA was synthesized from total RNA by RetroScript III reverse transcriptase (Invitrogen) with random primers. The cDNA was then subjected to PCR analysis with gene specific primers in the presence of SYBR green (Bio-Rad). Relative abundance of mRNA was obtained by normalization to 18S levels. The primers used for Bim qPCR are 5′-CACCATGGCA AGCAACCTTCTGATG-3′ (fwd) and 5′-TCAATGCATTCTCCACACC-3′ (rev). The software used to analyzed qPCR data is MxPro-Mx3000P v.4.10 Build 389, Schema 85 Stratagene.

### Cells and transfection

SH-SY5Y cells (ATCC) were transfected using Effectene transfection reagent (Qiagen). MPP^+^ (Sigma) treatment was performed according to Kalivendi et al. (2004).

### Caspase-3 activity assay

The Apo-Alert kit (Clontech) was used to measure caspase-3 activity according to manufacturer's protocol.

### Statistical analysis

The analysis was performed using Two-way ANOVA. The type of statistical analysis used is indicated in each figure legend. Significant differences are demonstrated by single symbols (∗, #, €, ∗∗∗) indicating *p* < 0.01, 0.001, 0.05. Error bars in figures represent s.e.m.

## Results and discussion

We wished to test whether the deletion of SIRT2 could rescue MPTP-induced nigrostriatal damage by using SIRT2 KO mouse model. SIRT2 KO animals show no differences in brain development and gross anatomy. Gross histological examination of the brain reveals normal morphology in SIRT2 KO mice (data not shown, 10). MPTP is the most commonly used dopaminergic neurotoxin that leads to reproducible nigrostriatal damage after systemic administration. It is accepted as a pharmacological model of Parkinson's disease (Dauer and Przedborski, 2003). The chronic MPTP regimen leads to neurodegeneration via apoptosis (Dauer and Przedborski, 2003). We thus administered MPTP i.p. (intraperitoneal injection) to SIRT2 KO and wild type (wt) mice via chronic regimen (Tatton and Kish, 1997; Jackson-Lewis and Przedborski, 2007). Nigrostriatal damage caused by the chronic regimen of MPTP is the death of dopaminergic neurons via apoptosis. The number of dopaminergic neurons in the substantia nigra pars compacta (SNpc) and the striatal fibers were assessed by TH (tyrosine-hydroxylase) immunoreactivity.

**Figure 1:**
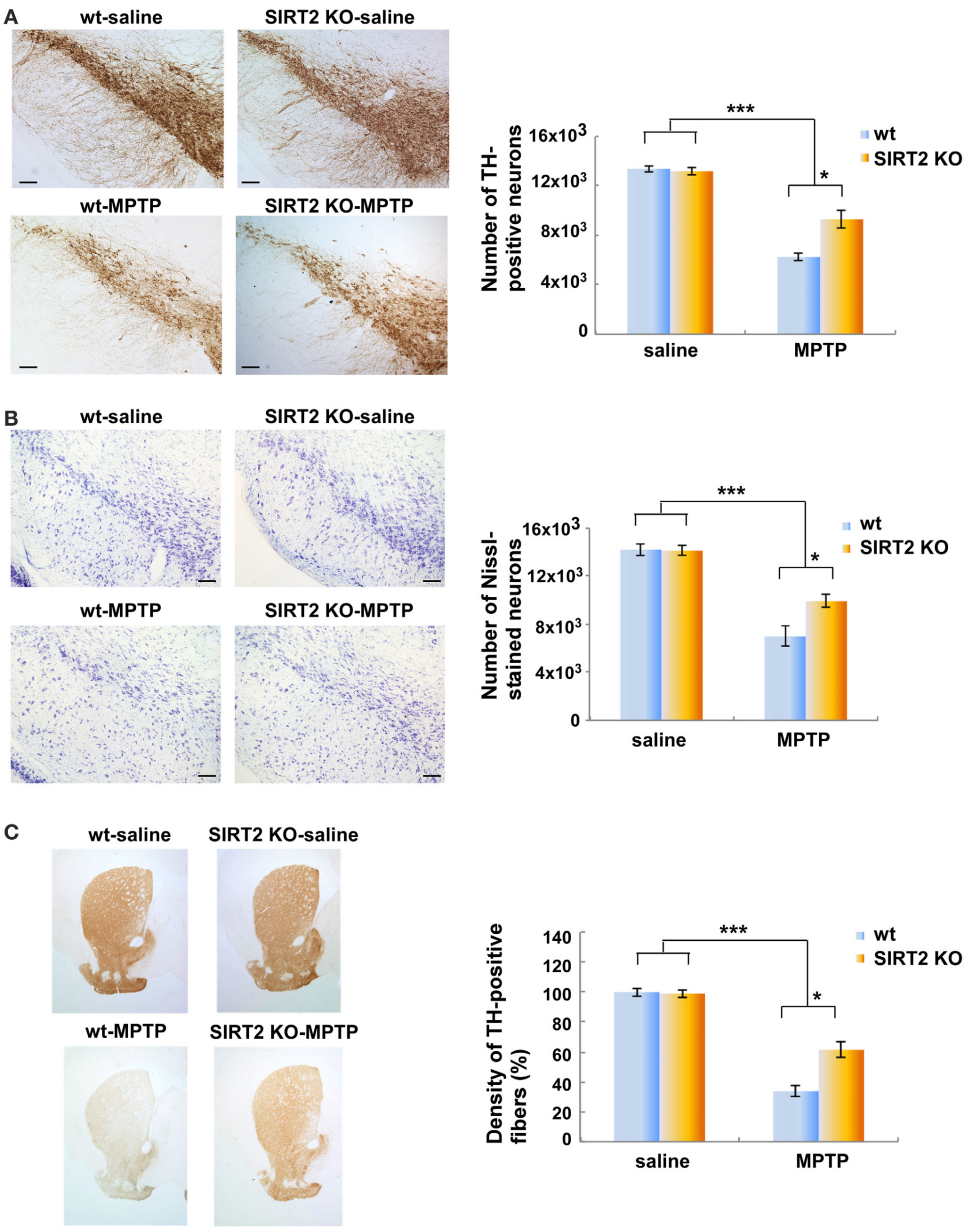
**Deletion of SIRT2 reduces the MPTP-induced nigrostriatal damage in mouse brains. (A)** The left panel shows immunostaining of TH-positive neurons in the substantia nigra pars compacta (SNpc) in saline- or MPTP-dosed wt or SIRT2 KO mice. Scale bar represents 100 μM. The quantification on the right shows the number of SNpc neurons counted by the image analysis tool of NIS-Elements AR microscope software. Bars represent s.e.m (*n* = 4–5). Statistical analyses were carried out using Two-Way ANOVA. ^***^p < 0.001, MPTP vs. saline groups; ^*^p < 0.05, SIRT2 KO-MPTP vs. wt-MPTP. **(B)** The left panel shows the Nissl staining of the neurons in SNpc of saline- or MPTP-dosed mice. Scale bar represents 100 μM. The quantification on the right shows the number of Nissl-stained neurons in SNpc counted by the image analysis tool of NIS-Elements AR microscope software. Bars represent s.e.m (*n* = 4–5). Statistical analyses were carried out using Two-Way ANOVA. ^***^p < 0.001, MPTP vs. saline groups; ^*^p < 0.05, SIRT2 KO-MPTP vs. wt-MPTP. **(C)** The left panel shows the TH-positive striatal fibers in saline- or MPTP-dosed wt or SIRT2 KO mice. Quantification on the right shows the loss of striatal fibers in MPTP-dosed mice assessed by optical density (*n* = 4–5). Statistical analyses were carried out using Two-Way ANOVA. ^***^p < 0.001, MPTP vs. saline groups; ^*^p < 0.05, SIRT2 KO-MPTP vs. wt-MPTP.

We observed a marked reduction in the number of TH-positive neurons in MPTP-treated wt or SIRT2 KO mice compared to saline-treated wt or SIRT2 KO mice (controls) (Figure 1A, *n* = 4–5 for each group). However, the number of TH-positive neurons in MPTP-treated SIRT2 KO mice was significantly higher than MPTP-treated wt mice. This result indicates that deletion of SIRT2 leads to a decrease in the nigrostriatal damage caused by MPTP (Figure 1A). We also analyzed the number of neurons in substantia nigra by Nissl staining and observed that it was reduced after MPTP treatment in wt mice (Figure 1B). However, the number of neurons in MPTP-treated SIRT2 KO mice was significantly higher than MPTP-treated wt mice (Figure 1B). Similarly, the density of TH-positive striatal fibers was reduced after MPTP-treatment in wt mice (Figure 1C). However, the density of TH-positive striatal fibers in MPTP-dosed SIRT2 KO mice was higher compared to the MPTP-dosed wt mice (Figure 1C). These data demonstrate that the deletion of SIRT2 prevents the loss of TH-positive neurons of SNpc and the striatal fibers after MPTP treatment.

**Figure 2:**
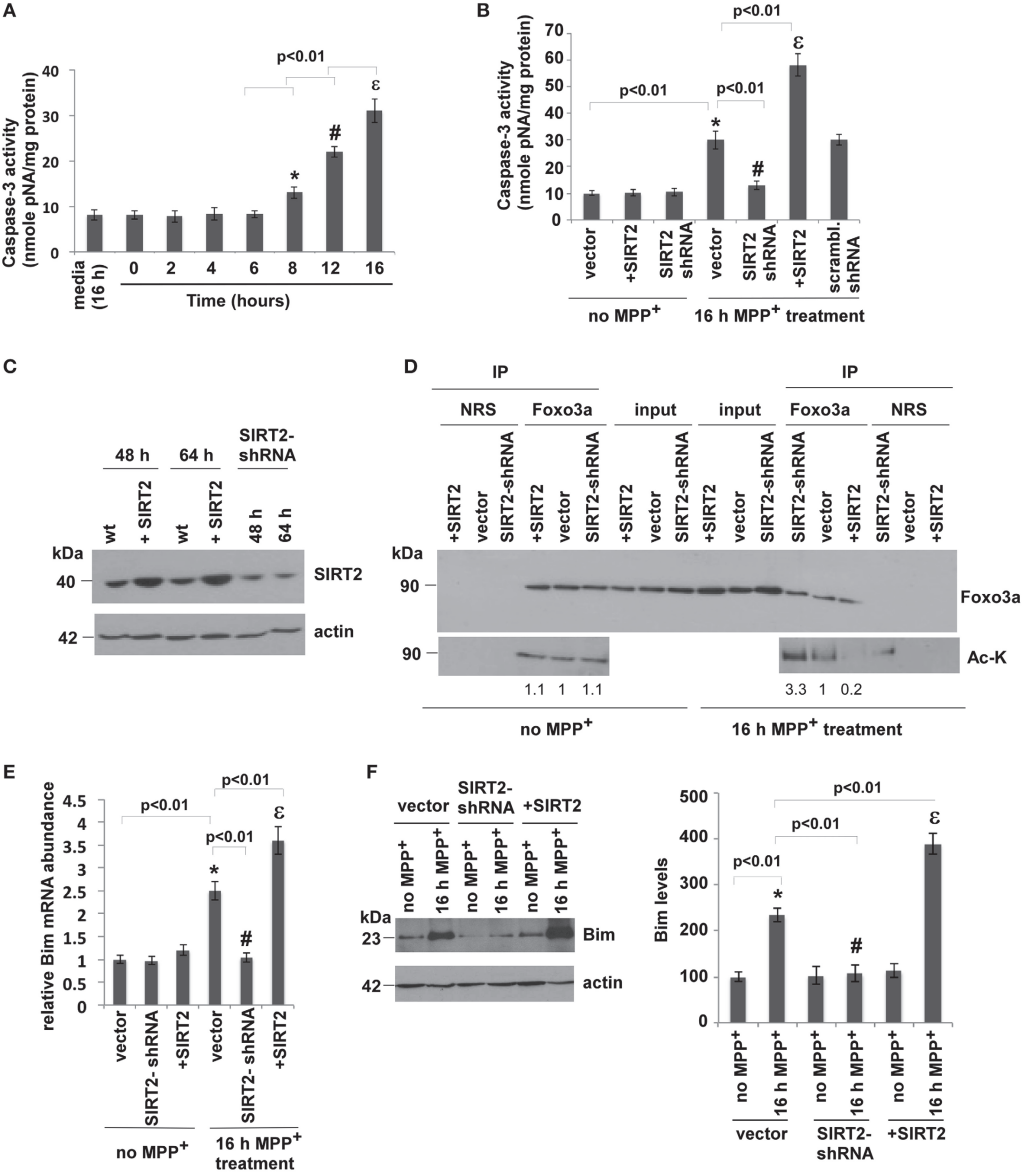
**SIRT2 deacetylates Foxo3a, increases Bim levels and leads to apoptosis in MPP^+^-treated cells. (A)** MPP^+^ treatment increases caspase-3 activity in SH-SY5Y (neuroblastoma) cells. Caspase-3 activity was determined from the supernatants of SH-SY5Y cell lysates after treatment with MPP^+^ for the indicated times. Bars represent s.e.m of three independent experiments. Statistical analyses were carried out using Two-way ANOVA. ^*^p < 0.01, 8 h vs. 0 h; ^#^*p* < 0.01, 12 h vs. 8 h; ^ε^*p* < 0.01, 16 h vs. 12 h. **(B)** Overexpression of SIRT2 increases and silencing SIRT2 decreases the caspase-3 activity that is elevated after MPP^+^ treatment. The graph shows the caspase-3 activity in the supernatants of SH-SY5Y cell lysates where SIRT2 is overexpressed (+SIRT2) or silenced (SIRT2-shRNA) without (no MPP^+^) or with MPP^+^ treatment for 16 h. Bars represent s.e.m of three independent experiments. Statistical analyses were carried out using Two-Way ANOVA. ^*^p < 0.01, vector with MPP^+^ vs. vector no MPP^+^; ^#^*p* < 0.01, SIRT2-shRNA with MPP^+^ vs. vector with MPP^+^; ^ε^*p* < 0.01, +SIRT2 with MPP^+^ vs. vector with MPP^+^. **(C)** Relative protein levels of SIRT2 are increased as a result of transfection with SIRT2 plasmid and reduced as a result of silencing with SIRT2-shRNA plasmid (total 48 h). The expressions were also measured after 64 h (48 h transfection + 16 h MPP^+^ treatment). The panel shows a representative western blot of SIRT2 levels from the lysates of SH-SY5Y cells transfected with SIRT2 (+SIRT2), or SIRT2-shRNA (SIRT2-shRNA) plasmids or empty vector (wt) with anti-SIRT2 antibody. Actin serves as a loading control. **(D)** SIRT2 deacetylates Foxo3a after MPP^+^ treatment. The panel shows the lysates of SH-SY5Y cells without MPP^+^ treatment transfected with SIRT2 (+SIRT2) or SIRT2-shRNA (SIRT2-shRNA) or empty vector (vector) immunoprecipitated with Foxo3a antibody or NRS (Normal Rabbit Serum) and blotted with anti-Foxo3a and anti-acetylated lysine (Ac-K) antibodies without or with 16 h of MPP^+^ treatment. Quantification of the acetylated bands was carried out by densitometry using the NIH ImageJ program and is shown below the gel. **(E)** Relative Bim RNA levels quantified by qPCR from SH-SY5Y cell extracts transfected with vector, SIRT2 or SIRT2-shRNA plasmid with or without MPP^+^ treatment. Bars represent s.e.m of three independent experiments. Statistical analyses were carried out using Two-Way ANOVA. ^*^p < 0.01, vector with MPP^+^ vs. vector no MPP^+^; ^#^*p* < 0.01, SIRT2-shRNA with MPP^+^ vs. vector with MPP^+^; ^ε^*p* < 0.01, +SIRT2 with MPP^+^ vs. vector with MPP^+^. **(F)** Western blotting of Bim protein extracted from the lysates of SH-SY5Y cells transfected with SIRT2 (+SIRT2) or SIRT2-shRNA (SIRT2-shRNA) or empty vector (vector) with or without MPP^+^ treatment. Quantification was carried out by densitometry using the NIH ImageJ program and is shown on the right. Three independent experiments were performed. Representative blot is shown. Statistical analyses were carried out using Two-Way ANOVA. ^*^p < 0.01, vector with 16 h MPP^+^ vs. vector no MPP^+^; ^#^*p* < 0.01, SIRT2-shRNA with 16 h MPP^+^ vs. vector with 16 h MPP^+^; ^ε^*p* < 0.01, +SIRT2 with MPP^+^ vs. vector with 16 h MPP^+^.

Since chronic administration of MPTP induces apoptotic neuronal death in mouse brains, we wanted to analyze whether silencing or overexpressing SIRT2 affects the MPP^+^-induced apoptosis in SH-SY5Y (neuroblastoma) cells. Therefore, we overexpressed or silenced SIRT2 in SH-SY5Y cells and assayed apoptosis. We used caspase-3 activity as a measure of MPP^+^-induced apoptosis. Caspase-3 is an active cell-death protease involved in the execution phase of apoptosis, where cells undergo morphological changes such as DNA fragmentation, chromatin condensation, and apoptotic body formation (Porter and Jänicke, 1999). Caspase-3 is activated in response to treatment with pharmacological agents such as MPP^+^. SH-SY5Y cells were treated with media alone or MPP^+^ to a final concentration of 500 μM for different time intervals (2, 4, 6, 8, 12, 16 h) (Kalivendi et al., 2004). Caspase-3 activity in cells treated 16 h with medium alone was comparable to the activity in untreated cells (0 h, Figure 2A). MPP^+^ treatment increased caspase-3 activity only after 8 h of treatment, reaching the highest levels after 16 h (Figure 2A).

To test the effect of SIRT2 on MPP^+^-induced apoptosis, SH-SY5Y cells were transfected with empty vector (wt), SIRT2 plasmid to overexpress SIRT2, or SIRT2-shRNA plasmid to silence SIRT2 (See Experimental Procedures). We first overexpressed or silenced SIRT2 in cells and checked SIRT2 levels both after transfection (48 h) and also after 16 h MPP^+^ treatment (totally 64 h) and confirmed overexpression or silencing in both cases (Figure 2C). 48 h after transfection, cells were treated with MPP^+^ for 16 h, and then caspase-3 activity was analyzed. SIRT2 overexpression or silencing had no effect on caspase-3 activity in the absence of MPP^+^ treatment (Figure 2B). After 16 h of MPP^+^ treatment, caspase-3 activity was increased in wt cells (vector, Figure 2B). SIRT2 silencing decreased caspase-3 activity to baseline while overexpressing SIRT2 increased caspase-3 activity. Scrambled shRNA did not have any effect on caspase-3 activity. This result indicates that silencing SIRT2 prevents MPP^+^-induced apoptosis in SH-SY5Y cells.

SIRT2 was shown to promote cell death when cells are under severe stress by activating Bim, a pro-apoptotic factor (Wang et al., 2007). It was shown that, in cell culture, SIRT2 deacetylates Foxo3a. Since Bim is a pro-apoptotic factor that is one of Foxo3a's target genes, we analyzed whether SIRT2 deacetylates Foxo3a in SH-SY5Y cells and elevates Bim expression. In order to measure the acetylation level of Foxo3a in MPP^+^-treated cells with SIRT2 overexpression or silencing, we immunoprecipitated Foxo3a from the extracts of cells transfected with control vector, SIRT2 plasmid or SIRT2 shRNA plasmid using Foxo3a antibody. We then blotted the eluates with acetylated lysine (Ac-K) antibody to detect the acetylation of Foxo3a (Figure 2D). The acetylation levels of Foxo3a were not changed in the absence of MPP^+^ treatment (no MPP^+^) where SIRT2 was silenced or overexpressed (Figure 2D, left panel). After 16 h MPP^+^ treatment, we observed that the acetylation levels of Foxo3a were decreased in SIRT2 overexpressing cells and increased in SIRT2-silenced cells compared to empty vector. This result demonstrates that SIRT2 deacetylates Foxo3a in SH-SY5Y cells only after MPP^+^ treatment (Figure 2D, right panel).

We then tested whether the deacetylation of Foxo3a by SIRT2 elevates the expression level of Bim in cells. We first analyzed the RNA levels of Bim (Figure 2E). After 16 h of MPP^+^ treatment, Bim RNA levels were increased with overexpressing SIRT2 and decreased with silencing SIRT2. There was no difference in Bim RNA levels with SIRT2 overexpression or silencing in the absence of MPP^+^ treatment (Figure 2E). The protein levels of Bim in cells were consistent with its RNA levels (Figure 2F).

**Figure 3:**
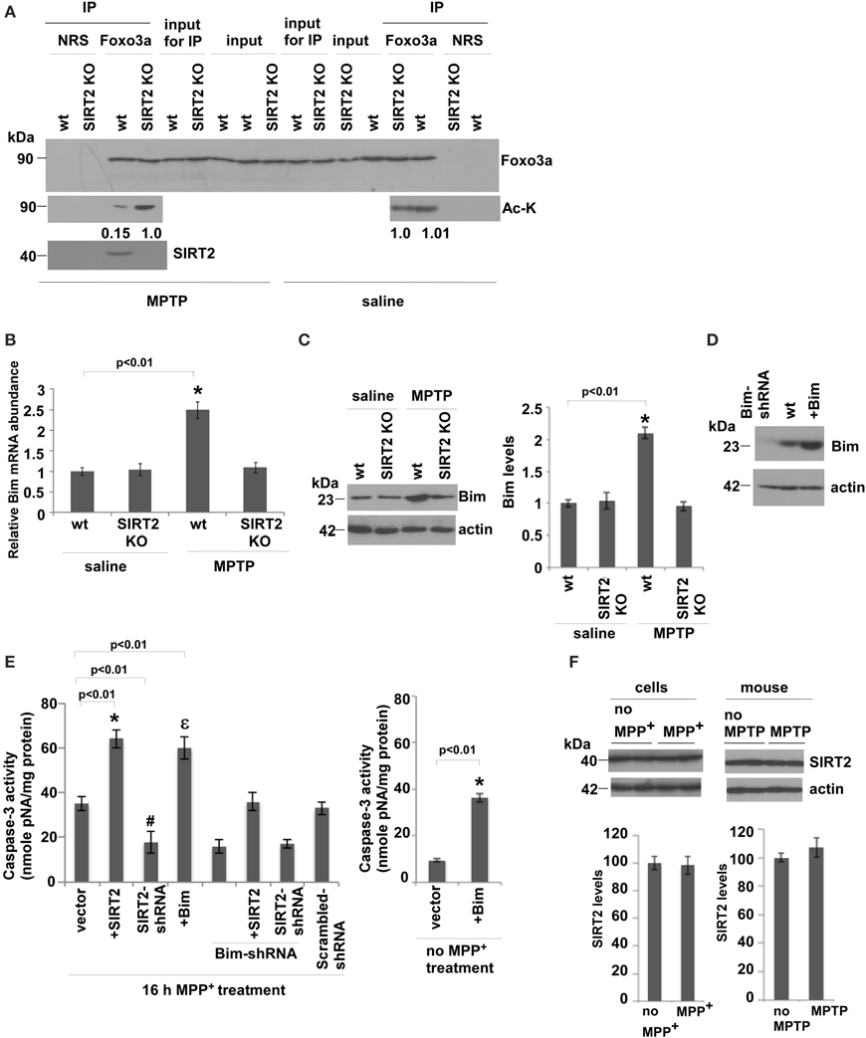
**SIRT2 deacetylates Foxo3a, increases Bim levels and leads to apoptosis in MPTP-treated mouse brains. (A)** SIRT2 deacetylates Foxo3a in MPTP-injected mouse brains. The lysates from wt or SIRT2 KO mouse brains were immunoprecipitated with Foxo3a antibody or NRS (Normal Rabbit Serum) and blotted with anti-Foxo3a or anti-acetylated lysine (Ac-K) antibodies in saline or MPTP-injected mouse brains. Quantification of acetylated bands was carried out by densitometry using the NIH ImageJ program and is shown below the gel. Representative blots are shown. **(B)** Bim RNA levels quantified from whole brains of mice by qPCR. *n* = 6 for each indicated group. Statistical analyses were carried out using Two-Way ANOVA. ^*^p < 0.01, wt-MPTP vs. wt-saline. **(C)** Western blotting of Bim protein extracted from whole brains of saline or MPTP-treated wt or SIRT2 KO mouse brains. *n* = 6 for each group. Actin serves as loading control. Representative immunoblot is shown. Quantification of the relative Bim protein levels is shown on the right. Statistical analyses were carried out using Two-Way ANOVA. ^*^p < 0.01, wt-MPTP vs. wt-saline. **(D)** Western blotting of Bim levels from the lysates of SH-SY5Y cells that are transfected with Bim or Bim-shRNA plasmids. Actin serves as a loading control. **(E)** Caspase-3 activity of SH-SY5Y cells transfected with SIRT2, SIRT2-shRNA, Bim or Bim-shRNA plasmids and treated with 16 h MPP^+^. The graph on the right shows Caspase-3 activity of SH-SY5Y cells after Bim overexpression without MPP^+^ treatment. **(F)** Left panel shows the western blotting of SIRT2 protein extracted from wt or MPP^+^-treated SH-SY5Y cells. Three independent experiments were performed. Right panel shows the western blotting of SIRT2 protein extracted from whole brains of saline or MPTP-treated wt mice. *n* = 6 for each group. Actin serves as loading control. Representative immunoblots are shown. Quantifications of the relative SIRT2 protein levels are shown below. Statistical analyses were carried out using Two-Way ANOVA.

We then analyzed whether SIRT2 deacetylates Foxo3a and increases Bim levels in saline or MPTP-injected mouse brains. We observed that acetylation levels of Foxo3a increase only in MPTP-treated SIRT2 KO mouse brains compared to wt brains, indicating that SIRT2 deacetylates Foxo3a only after MPTP treatment (Figure 3A). To investigate whether Bim expression increases as a result of Foxo3a deacetylation, we analyzed the RNA levels of Bim in saline or MPTP-treated wt or SIRT2 KO mouse brains (Figure 3B) (*n* = 6). We observed that there is no difference in Bim RNA levels in saline treated wt or SIRT2 KO mice. However, Bim RNA levels increase in MPTP-treated wt mice brains, and not in MPTP-treated SIRT2 KO mice (Figure 3B). Bim protein levels were also consistent with RNA levels *in vivo*, increasing only in MPTP-treated wt mouse brains (Figure 3C) (*n* = 6). These data show that SIRT2 deacetylates Foxo3a and increases Bim levels only after MPTP treatment in mouse brains.

In order to confirm the mechanism that the increase in apoptosis after MPTP treatment is through the increase in Foxo3a deacetylation and Bim expression, we assayed caspase-3 activity in MPP^+^-treated SH-SY5Y cells where we manipulated the levels of SIRT2 and Bim (Figure 3E). Figure 3D indicates the protein levels of Bim after its silencing or overexpression in cells. Caspase-3 activity was increased when SIRT2 was overexpressed, and decreased when SIRT2 was silenced (Figure 3E). Overexpressing Bim increased caspase-3 activity to similar levels as in SIRT2 overexpression. In addition, silencing Bim decreased caspase-3 activity to similar levels as in SIRT2 silencing. Importantly, when we overexpress SIRT2 in MPP^+^-treated Bim-silenced cells, Caspase-3 activity was at the levels of wt cells (vector) and not as high as in the case of SIRT2 overexpression. This indicates that elevated Bim expression is the main cause of apoptosis. In addition, caspase-3 activity in cells expressing both SIRT2-shRNA and Bim-shRNA was not significantly lower than in cells expressing SIRT2-shRNA or Bim-shRNA alone indicating that the effect is not additive. The latter experiment suggests that SIRT2 and Foxo3a function in the same pathway to elevate caspase-3 activity. Scrambled-shRNAs did not have any effect on the caspase-3 activity (Figure 3E). In addition, the graph on the right shows that caspase-3 activity increases as a result of Bim overexpression in cells without MPP^+^ treatment (Figure 3E). We have also analyzed whether the expression level of SIRT2 is upregulated in MPP^+^-treated cells or MPTP-treated mice by western blotting (Figure 3F). We did not observe any change in SIRT2 protein levels in MPP^+^-treated cells compared to control cells or in MPTP-injected mice compared to control mice, indicating that the increase in Bim expression levels is not caused by the increase in SIRT2 expression (Figure 3F).

These data show that SIRT2 leads to neurodegeneration in MPTP-injected mice and MPP^+^-treated cells by deacetylating Foxo3a and increasing Bim levels, therefore leading to apoptosis. We also show that deletion of SIRT2 prevents neuronal death in MPTP-treated mice. Similarly, silencing SIRT2 in MPP^+^-treated cells also inhibits apoptosis. The results shown here are also consistent with the fact that sirtuins are stress-response genes. In this study, SIRT2 is shown to deacetylate Foxo3a and increase Bim levels only after MPP^+^-treatment in cells or MPTP-injection in mice. Similarly, in a previous study (Donmez et al., 2012), SIRT1 was shown to deacetylate HSF1 and increase Hsp70 levels only after heat shock in cells or only in the brains of Parkinson's disease mouse model (A53T alpha-synuclein). These studies show that SIRT1 and SIRT2 are activated as a result of stress conditions and turn on their target pathways. Therefore, in the future, designing SIRT2 inhibitors that become activated as a result of the disease condition might be useful in developing treatments for Parkinson's disease.

### Conflict of interest statement

The authors declare that the research was conducted in the absence of any commercial or financial relationships that could be construed as a potential conflict of interest.

## Supplementary material

Supplementary Figure 1 **(A–E)** Show various SIRT2 antibodies tested for specificity using two wt and two SIRT2 KO whole brain extracts of mice. Tubulin serves as a loading control and shown in the stripped gels below.

## Abbreviations

TH: tyrosine hydroxylase
SNpc: substantia nigra pars compacta
MPTP: 1-methyl-4-phenyl-1,2,3,6-tetrahydropyridine
SIRT2: Sirtuin2
KO: knockout.
