# Supplementary figures and images for: SIRT2 enhances 1-methyl-4-phenyl-1,2,3,6-tetrahydropyridine (MPTP)-induced nigrostriatal damage via apoptotic pathway
Source: Front Aging Neurosci. 2014 Aug 11;6:184. doi: 10.3389/fnagi.2014.00184 (PMC4127494; doi:10.3389/fnagi.2014.00184)

Supplementary Figure 1

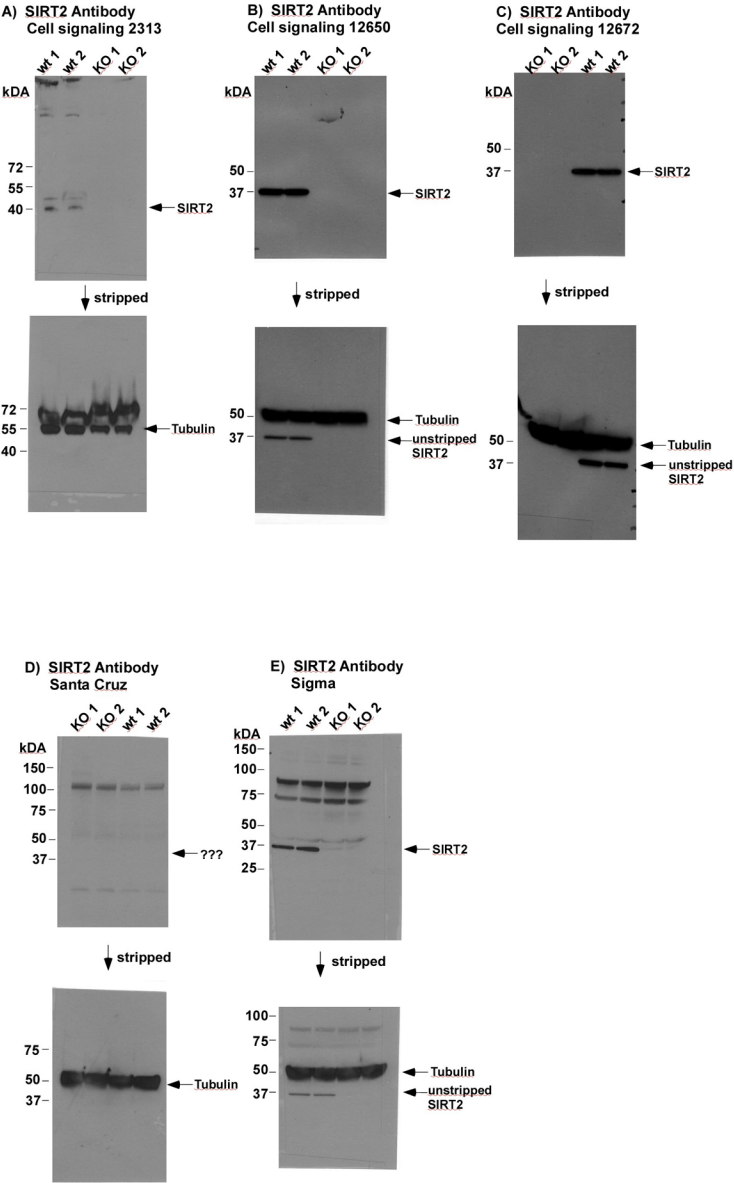

Supplement: Supplementary Figure 1 — (A–E) Show various SIRT2 antibodies tested for specificity using two wt and two SIRT2 KO whole brain extracts of mice. Tubulin serves as a loading control and shown in the stripped gels below. [file Presentation1.PDF]
